# Validation and Factor Structure of the French-Language Version of the Emotional Appetite Questionnaire (EMAQ)

**DOI:** 10.3389/fpsyg.2017.00442

**Published:** 2017-03-23

**Authors:** Léna Bourdier, Christophe Lalanne, Yannick Morvan, Laurence Kern, Lucia Romo, Sylvie Berthoz

**Affiliations:** ^1^EA 4430, CLIPSYD, University Paris NanterreNanterre, France; ^2^EA 7334 (REMES), Paris Sorbonne Cité, Patient-Centered Outcomes Research, University Paris DiderotParis, France; ^3^Unité de Recherche Clinique en Economie de la Santé, Hôpital Hôtel-Dieu, URC ECOParis, France; ^4^INSERM U894 CPN, LPMP, SHU, Centre Hospitalier Sainte AnneParis, France; ^5^EA2931 CERSM, UFR-STAPS, University Paris NanterreNanterre, France; ^6^INSERM U894 CPN, CMME Centre Hospitalier Sainte AnneParis, France; ^7^CESP, INSERM, University Paris-Sud, UVSQ, University Paris-SaclayVillejuif, France; ^8^Psychiatry Unit, Institut Mutualiste MontsourisParis, France

**Keywords:** emotional eating, scale validity, students, Body Mass Index, bulimia nervosa

## Abstract

The concept of Emotional Eating (EE) is increasingly considered to be implicated in overeating and obesity, and in different subtypes of eating disorders. Among the self-report questionnaires assessing EE, the Emotional Appetite Questionnaire (EMAQ) includes recent advances in this area: it evaluates a broad range of emotions and situations both positive and negative, and the way they modulate food intake (decrease, stability, or increase). The main objective of our study was to further investigate the psychometric properties of the French version of the EMAQ in a large sample of students. Participants completed the EMAQ (*n* = 679), the DEBQ (Dutch Eating Behavior Questionnaire) (*n* = 75) and the CIDI-eating disorders screening (Composite International Diagnostic Interview) (*n* = 604). Factorial structure, reliability, and validity of the EMAQ were tested. Factorial analyses supported a two-factor (Positive and Negative) structure. The internal consistency indices were satisfactory and results suggest good test–retest reliability for the scale. Convergent and discriminant validity were confirmed from the significant correlations observed between the EMAQ scores and the DEBQ-EE subscale scores. Regarding associations with weight, whereas EMAQ negative scores were positively correlated with BMI, EMAQ positive scores were negatively correlated with BMI. Finally, EMAQ scores differed significantly depending on gender and risk for bulimia nervosa. This study supports the validity and the reliability of the EMAQ, which appears to be a promising instrument to better understand individual differences that could modulate food intake.

## Introduction

Food consumption is considered an important mood regulating behavior (Heatherton and Baumeister, 1991; Polivy and Herman, 1993; Greeno and Wing, 1994; Macht, 1999, 2008). In this setting, Emotional Eating (EE) is generally conceived as eating in response to negative emotions rather than to feelings of hunger or satiety (Arnow et al., 1995; Lindeman and Stark, 2001). Indeed, some individuals appear to be more susceptible to unhealthy shifts in food choices (e.g., abnormal consumption of sweet, salty, high-fat, and energy-dense foods) in order to cope with negative emotions (Oliver et al., 2000; Nguyen-Michel et al., 2007; Macht, 2008; van Strien et al., 2012). Past research has shown that this eating behavior could place the individual at risk for overweight and obesity (Macht, 2008; Gibson, 2012; Singh, 2014). Moreover, both experimental and large scale epidemiological studies show that healthy and normal-weight persons use food to regulate negative emotions (Macht and Simons, 2000; Macht et al., 2005; Spoor et al., 2007; Camilleri et al., 2014; Finch and Tomiyama, 2015)^1^, which suggests that EE could be considered as a common phenomenon occurring in the general population. For instance, in a recent American survey among adults, 38% reported overeating unhealthy foods in the past month because of stress, and half of them reported engaging in these behaviors weekly or more (American Psychological Association, 2015). In a French national survey, 44.4% of respondents reported eating more under stress (Beck et al., 2007). Overeating is not the only stress-induced eating behavior. While few people report not changing their eating behaviors during stressful periods, there seem to be as many people who eat more (around 30–50% would present this a-typical stress response) as individuals who eat less (40–70% would present this typical distress response) (see Gibson, 2012). This disparity in either overeating or under-eating in the general population has been observed in clinical samples, and recent studies suggest that, besides obesity, EE could be involved in the entire spectrum of eating disorders: not only in binge eating episodes as in bulimia nervosa (BN) or binge eating disorder, but also in binge-purging and restrictive anorexia nervosa (Ricca et al., 2009, 2012). There is also growing evidence showing that positive emotions are considered to be an underestimated risk factor for food intake and overeating (Bongers et al., 2013a,b, 2016; Evers et al., 2013), but little is known about their effects on eating behaviors compared to those documented for negative emotions (Macht, 2008). However, recent studies suggest that eating in response to negative and to positive emotions could refer to different constructs (van Strien et al., 2013) and that only the desire to eat in response to negative emotions would be an ‘obese’ eating style (van Strien et al., 2016).

Several self-report questionnaires have been developed to assess EE. The first and most commonly used questionnaires are the Three-Factor Eating Questionnaire (TFEQ) (Stunkard and Messick, 1985), the Dutch Eating Behavior Questionnaire (DEBQ) (van Strien et al., 1986) and the Emotional Eating Scale (EES) (Arnow et al., 1995)^2^. The TFEQ (Disinhibition subscale) and the TFEQ Revised 18-items version (EE subscale) (Karlsson et al., 2000), comprise only three items assessing increased food intake in response to negative emotions. The second revised version (TFEQ-R21) introduced three supplementary items in the EE subscale (Cappelleri et al., 2009). The DEBQ (van Strien et al., 1986) is a multidimensional instrument (see Materials and Methods) that includes a 13-items EE subscale, with questions assessing increased food intake in response to negative emotions. The EES (Arnow et al., 1995) evaluates the desire or urge to eat in response to 25 specific negative emotional states. The EES has recently been extended to positive emotions and the instructions have been modified to also evaluate decreased intake (EES-II, Kenardy et al., 2003), but this version has not been validated as such (no factorial, reliability, or validity analysis).

In summary, these instruments are not fully suited to measuring the complexity of the effects of these emotionally driven eating behaviors, i.e., the fact that food intake can either increase or decrease in response to negative as well as positive emotions.

Geliebter and Aversa (2003) developed the Emotional Appetite Questionnaire (EMAQ), a self-report questionnaire based on ratings of the tendency to eat less, equally or more in response to both positive and negative primary emotions. In comparison with the EES-II, the EMAQ also explores commonly encountered situations. Geliebter and Aversa (2003) showed among young adults the internal consistency and the reliability of the scale, as well as its sensitivity to BMI categories and gender. Later, Nolan et al. (2010) demonstrated in a convenience sample of young adults (*n* = 232 university students or employees; 73.71% of women) the construct validity of the EMAQ (convergent and discriminant with the DEBQ-EE subscale) and replicated Geliebter and Aversa (2003) findings on BMI and gender. However, neither studies tested the proposed four-factor structure of the scale (based on the presence of four subscores: positive emotions, negative emotions, positive situations, negative situations) and evidence of the EMAQ clinical sensitivity is limited to the demonstration that overweight individuals report higher scores than normal-weight and underweight individuals.

Hence, although the EMAQ has the potential advantage to take account of different factors that are now known to modulate food intake and so to be a useful screening tool for the assessment of emotionally driven, non-homeostasic eating behaviors, it needs to be further validated.

The aim of the present study was to examine the psychometric properties of the French version of the EMAQ (F-EMAQ) in a large sample of students, as this population is considered at risk for developing EE. The students’ financial constraints can impact the healthiness of their diet (Gibson, 2012). Several studies showed that students’ weight (Anderson et al., 2003) and perceived stress (Tavolacci et al., 2013) increase, while at the same time physical activities decrease (Boujut and Koleck, 2009; Kern et al., 2013). Exam periods have been associated with an increased tendency to eat, with higher energy intake and a less healthy diet (Epel et al., 2004; Macht et al., 2005; Barker et al., 2015).

The study was designed to examine the factor structure, the internal consistency and the test–retest reliability of the F-EMAQ. Like in the Nolan et al. (2010) study, its convergent and discriminant validity were tested with the DEBQ-EE. Furthermore, we explored whether EMAQ scores discriminate individuals with versus without a risk for BN.

## Materials and Methods

This study was carried out in accordance with the recommendations of the Department of Psychological and Educational Sciences UFR-SPSE, University of Paris Nanterre ethics committee. All subjects gave written informed consent in accordance with the Declaration of Helsinki. The first page of the questionnaire booklet included the information and consent form. The participation was voluntary and anonymous. The data were collected during students’ lectures or tutorials in restricted groups.

### Participants

Two samples were used (**Table 1**): Sample 1 for the analyses of the EMAQ factorial structure and its associations with the risk for being diagnosed with disordered eating behaviors; Sample 2 for the analyses of the scale test–retest reliability and convergent and discriminant validity.

**Table 1:** Summary table of measures and statistical analyses for Sample 1 and Sample 2.

	Sample 1 (*n* = 604)	Sample 2 (*n* = 75)
Measures	Socio-demographic information	Socio-demographic information
	BMI	BMI
	EMAQ	EMAQ
	CIDI	DEBQ
Statistical analysis	Factor structure *(EFA, CFA)*	Test–retest reliability *(ICCs)*
	Internal consistency *(Pearson correlations, Cronbach’s Alpha)*	Convergent and discriminant validity *(Spearman correlations)*
	Association with BMI *(Spearman correlations)*	-
	Between-group comparisons *(ANCOVA)*	-

*BMI, Body Mass Index (kg/m^2^); EMAQ, Emotional Appetite Questionnaire; CIDI, Composite International Diagnostic Interview; DEBQ, Dutch Eating Behavior Questionnaire; EFA, Exploratory Factor Analysis; CFA, Confirmatory Factor Analysis; ANCOVA, analysis of covariance (BMI-adjusted); ICCs, Intra-Class Correlation coefficients.*

Sample 1 was derived from a survey on students’ physical and mental health (*n* = 750 students from the University of Paris Nanterre). Inclusion criteria for the present analyses were: 18 years old or more, with the EMAQ and the questionnaire assessing the risk of eating disorders fully completed. Sample 2 comprised 75 students in Psychology (Ecole des Psychologues Praticiens). These participants completed the EMAQ and DEBQ questionnaires. In addition, they completed the EMAQ a second time, at an interval of 4–6 weeks.

### Measures

Socio-demographic information was collected (age, gender, academic discipline, level of education) as well as self-reported height and weight.

#### Emotional Appetite Questionnaire (EMAQ) (Geliebter and Aversa, 2003; Nolan et al., 2010)

The EMAQ contains 22 items assessing variations of food intake in response to emotional states and situations: nine items evaluate negative emotions (e.g., “*When you are sad”*), five evaluate positive emotions (e.g., *“When you are happy”*), five refer to negative situations (e.g., “*When under pressure”*) and three to positive situations (e.g., *“When engaged in an enjoyable hobby”).* On a nine point Likert-type scale, for each item respondents were asked to rate whether they ate less (from 1 to 4), the same (5), or more (from 6 to 9) compared to usual. Two optional responses (not included in the scoring) are also possible: Not applicable or Don’t know. A score is calculated for positive emotions (EMAQ-PE), negative emotions (EMAQ-NE), positive situations (EMAQ-PS) and negative situations (EMAQ-NS). A positive total score (EMAQ-P) is obtained by averaging the EMAQ-PE and EMAQ-PS scores. A negative total score (EMAQ-N) is obtained by averaging the EMAQ-NE and EMAQ-NS scores. In the two previous validation studies (Geliebter and Aversa, 2003; Nolan et al., 2010), Cronbach’s alphas were respectively 0.78 and 0.79 for EMAQ-NE, 0.75 and 0.87 for EMAQ-PE, 0.65 and 0.75 for EMAQ-NS, 0.57 and 0.66 for EMAQ-PS. Cronbach’s alphas for the total Positive and Negative scores were not reported.

The French adaptation of the EMAQ was conducted following the standard procedure. The authors of the original version of the EMAQ gave their agreement (to SB) for the adaptation into French of this instrument. The EMAQ was translated into French independently by four senior researchers (SB, YM, LK, and LR). These different translations were then compared and for the few items for which the exact wording differed (one positive situation and one negative situation), the final wording was based on consensus. This version was then back-translated by an English native speaker with expertise in psychology (postdoctoral fellow at CESP-INSERM). This version was compared to the original EMAQ. Only one item was slightly different (negative situation: ‘After a heated argument’): since in French this can refer to either a quarrel or a discussion, these two slightly different meanings of the word ‘argument’ were kept in the final French version (available on request from the authors).

#### Dutch Eating Behavior Questionnaire (DEBQ) (van Strien et al., 1986; French version: Lluch et al., 1996)

The DEBQ contains three subscales: Restrained Eating (DEBQ-R), External Eating (DEBQ-X) and Emotional Eating (DEBQ-EE). The DEBQ-EE has 13 items assessing eating more in response to negative emotions on a five-point Likert scale (from “never” to “very often”). Here, only DEBQ-EE scores were used (Cronbach alpha = 0.89).

#### Composite International Diagnostic Interview (CIDI) (World Health Organization, 1990)

This is a diagnostic structured instrument designed to estimate the presence of mental disorders. Five “diagnostic entry” questions assess, with a four-point self-rating scale, the risk for BN and/or anorexia nervosa according to three different temporal criteria: present risk, risk in the past year, lifetime risk. Here, we used the scoring for the lifetime risk for BN.

### Statistical Methods

Regarding descriptive statistics, continuous variables were summarized using mean, standard deviation and range, while categorical variables were summarized as counts and proportions. Two-group comparisons were performed using Student’s *t*-tests, while Pearson’s chi-squared tests were used to analyze cross-classification tables.

The descriptive statistics for the EMAQ items (i.e., response distribution across the nine response categories and ceiling/floor effects) were computed on Sample 1. To assess the factor structure of the EMAQ, Sample 1 was divided into two equal-sized subsamples, stratified by terciles for age and gender. A training sample was used to extract an interpretable factor structure using exploratory factor analysis (EFA) with Mplus default ML estimator for continuous outcomes, and a confirmatory (CFA) model for the same factor structure was tested on the validation subsample. The same confirmatory analysis was replicated following inspection of modification indices by including correlated errors. Besides the χ^2^ of model fit, standard fit indices (Hu and Bentler, 1999; Jackson et al., 2009) were used to assess goodness-of-fit of the CFA model: a comparative fit index (CFI) greater than 0.90; a standardized root mean square residual (SRMR) below 0.10; a root mean square error of approximation (RMSEA) below 0.08. Based on the factor structure determined during exploratory and confirmatory approaches on the training/validation subsamples, a final CFA model was applied to the full sample.

Internal structure and consistency were evaluated using Pearson correlations and Cronbach’s Alpha respectively. Test–retest stability was assessed using intra-class correlation coefficients. Spearman correlations were used to evaluate monotonic associations of EMAQ scores with DEBQ scores and BMI. Group comparisons for Gender and Diagnosis (at risk or not for lifetime BN) on EMAQ-P and EMAQ-N scores were estimated by analyses of variance and associated effect sizes (partial eta-squared, η_p_^2^). A value of η_p_^2^ around 0.01 is associated with a small effect, around 0.06 with a medium effect, and around 0.14 with a large effect (Cohen, 1988).

Exploratory factor analysis and CFA were carried out using R 2.15.2 (R Core Team, 2014) and Mplus 7 (Muthén and Muthén, 2013) software. The remaining analyses were carried out using SPSS 20.0 (Ibm Corp. Released, 2011). A fixed Type I error rate of 5% was retained for all statistical tests.

## Results

### Samples Characteristics

The characteristics of the study participants are summarized in **Table 2**.

**Table 2:** Descriptive statistics for Sample 1 and Sample 2 characteristics.

		Sample 1 (*n* = 604)	Sample 2 (*n* = 75)
Age (years)		21.3 ± 4.57 [18–59]	22.6 ± 4.27 [18–46]
Gender	Men	242 (40%)	5 (7%)
	Women	362 (60%)	70 (93%)
BMI (kg/m^2^)		21.8 ± 3.25 [15.4–39.5]	21.9 ± 3.06 [16.5–35.1]
Level of education	L1	34%	26%
	L2	8%	7%
	L3	51%	22%
	M1	6%	20%
	M2	1%	25%
Discipline	Psychology	48%	100%
	STAPS	43%	.
	Other	8%	.

*BMI, Body Mass Index (kg/m^2^); L1-L2-L3, degree year; M1–M2, masters level; STAPS, physical and sports activities faculty.*

Sample 1 included 604 students fulfilling the inclusion criteria. The men presented higher BMI (Mean = 22.3; *SD* = 2.6) than the women (Mean = 21.4; *SD* = 3.5, *p* = 0.002). Regarding academic discipline, 68% of the women were studying Psychology and 76% of the men were STAPS students (physical and sports activities faculty).

Sample 2 included 75 students. This sample differed from Sample 1 in terms of gender (93% women, *p* < 0.001) and age (Mean = 22.6; *SD* = 4.3, *p* = 0.020) but had similar BMI (Mean = 21.9; *SD* = 3.1, *p* = 0.757).

### Factor Structure (Sample 1)

The analysis of the distribution of responses to each item revealed no evidence of ceiling or floor effect: the highest proportion of responses in either of the two extreme response categories did not exceed 45%. The proportion of Not Applicable and Don’t know responses per item was less than 1.6%, except for item 13 ‘*Lonely*’ (1.8%).

Two to four factor solutions were assessed using EFA with PROMAX rotation on the training subsample (*n* = 302). A two-factor structure unambiguously reflecting two major dimensions was found: positive (EMAQ-P, eight items) and negative (EMAQ-N, 14 items) valence. Only one item was found to load weakly on its hypothetical factor (‘*Bored*,’ λ = 0.176) (see **Table 3**). There was no evidence of item cross-loadings, and the RMSEA for this model was estimated at 0.094 (90% CI[0.086; 0.101]).

**Table 3:** Two to four factor solutions of the Exploratory Factor Analysis (EFA on the training subsample, *n* = 302).

Items	Description	Two factor solution		Three factor solution			Four factor solution			
		1	2	1	2	3	1	2	3	4
1	Sad	**0.728**	0.142	**0.523**	**0.565**	0.031	**0.697**	0.196	0.041	0.196
2	Bored	**0.176**	-0.005	0.020	**0.349**	-0.079	0.042	**0.668**	-0.103	0.034
3^∗^	Confident	-0.073	-**0.649**	0.056	-0.162	-**0.621**	-0.040	0.103	-**0.633**	-0.305
4	Angry	**0.694**	0.022	**0.590**	0.311	-0.043	**0.702**	-0.017	-0.043	0.074
5	Anxious	**0.654**	0.081	**0.597**	0.187	0.039	**0.650**	-0.008	0.037	-0.048
6^∗^	Happy	-0.144	-**0.739**	0.004	-0.198	-**0.707**	-0.082	0.016	-**0.713**	-0.325
7	Frustrated	**0.647**	0.168	**0.496**	**0.376**	0.092	**0.635**	0.018	0.098	0.167
8	Tired	**0.455**	-0.111	**0.425**	0.142	-0.142	**0.468**	0.009	-0.153	-0.030
9	Depressed	**0.776**	0.177	**0.604**	**0.494**	0.076	**0.736**	0.227	0.080	0.095
10	Frightened	**0.478**	-0.021	**0.458**	0.100	-0.041	**0.500**	-0.103	-0.048	-0.021
11^∗^	Relaxed	-0.064	-**0.712**	0.079	-0.181	-**0.683**	-0.034	0.145	-**0.696**	-0.433
12^∗^	Playful	-0.107	-**0.760**	-0.095	0.089	-**0.781**	-0.045	0.006	-**0.768**	-0019
13	Lonely	**0.547**	0.081	**0.415**	**0.350**	0.011	**0.461**	**0.525**	0.003	-0.084
14^∗^	Enthusiastic	-0.123	-**0.886**	-0.064	0.012	-**0.889**	-0.047	-0.008	-**0.878**	-0.111
15	When under pressure	**0.723**	0.047	**0.636**	0.271	-0.012	**0.698**	0.116	-0.014	-0.053
16	After a heated argument	**0.676**	-0.053	**0.783**	-0.060	-0.048	**0.721**	-0.063	-0.052	-0.376
17	After a tragedy of someone close to you	**0.580**	0.037	**0.724**	-0.154	0.067	**0.632**	-0.138	0.066	-0.355
18^∗^	When falling in love	0.091	-**0.321**	0.063	0.134	-**0.346**	0.143	-0.097	-**0.352**	0.132
19	After ending a relationship	**0.634**	0.134	**0.616**	0.149	0.101	**0.640**	-0.005	0.111	-0.130
20^∗^	When engaged in an enjoyable hobby	-0.004	-**0.487**	0.030	0.011	-**0.487**	0.082	-0.190	-**0.496**	0.054
21	After losing money or property	**0.436**	-0.090	**0.559**	-0.161	-0.050	**0.466**	-0.063	-0.056	-**0.351**
22^∗^	After receiving good news	-0.027	-**0.644**	-0.064	0.178	-**0.688**	0.032	-0.008	-**0.700**	0.136

*^∗^Positive items. The highest loadings are hightlighted in bold.*

The CFA model for this bifactorial structure yielded model fit indices in the acceptable range (RMSEA = 0.089, 90% CI[0.082; 0.096]; CFI = 0.803; SRMR = 0.080) on the validation subsample (*n* = 302). Following analysis of modification indices, a second CFA model was tested on this validation sample by allowing items 16 and 17 to have correlated errors, but without much improvement in overall goodness-of-fit (RMSEA = 0.086, 90% CI[0.079; 0.093]; CFI = 0.818; SRMR = 0.079).

Based on the full sample (*n* = 604; see **Table 4** for the factor loadings), the final goodness-of-fit indices were: χ^2^(208; *n* = 604) = 686.98, *p* < 0.000; RMSEA = 0.079, 90% CI[0.074; 0.084]; CFI = 0.832; SRMR = 0.067.

**Table 4:** Two-factor solution and item factor loadings of the French-EMAQ (Confirmatory Factor Analysis – CFA on the full sample, *n* = 604).

		Factor 1		Factor 2	
Items	Description	Unstandardized loadings (*SE*)	Standardized loadings (*SE*)	Unstandardized loadings (*SE*)	Standardized loadings (*SE*)
3^∗^	Confident	0.271 (0.023)	0.501 (0.035)		
6^∗^	Happy	0.396 (0.024)	0.662 (0.028)		
11^∗^	Relaxed	0.320 (0.022)	0.594 (0.031)		
12^∗^	Playful	0.443 (0.021)	0.777 (0.022)		
14^∗^	Enthusiastic	0.403 (0.019)	0.786 (0.021)		
18^∗^	When falling in love	0.237 (0.031)	0.344 (0.041)		
20^∗^	When engaged in an enjoyable hobby	0.269 (0.028)	0.412 (0.038)		
22^∗^	After receiving good news	0.307 (0.021)	0.603 (0.031)		
1	Sad			0.560 (0.033)	0.661 (0.028)
2	Bored			0.084 (0.025)	0.148 (0.044)
4	Angry			0.400 (0.030)	0.552 (0.033)
5	Anxious			0.503 (0.035)	0.590 (0.031)
7	Frustrated			0.463 (0.032)	0.592 (0.031)
8	Tired			0.179 (0.032)	0.244 (0.042)
9	Depressed			0.585 (0.033)	0.692 (0.026)
10	Frightened			0.236 (0.028)	0.373 (0.040)
13	Lonely			0.417 (0.034)	0.519 (0.035)
15	When under pressure			0.502 (0.033)	0.604 (0.030)
16	After a heated argument			0.423 (0.029)	0.601 (0.031)
17	After a tragedy of someone close to you			0.326 (0.026)	0.551 (0.034)
19	After ending a relationship			0.453 (0.032)	0.590 (0.032)
21	After losing money or property			0.236 (0.027)	0.392 (0.040)

*^∗^Positive items. All loadings are significant at *p* < 0.000 except for item 2 (*p* = 0.001). *SE*, standard error.*

For the remaining analyses, besides EMAQ-P and EMAQ-N scores, we report but do not discuss the results for the EMAQ subscales in order to compare our results with those of the two previous validation studies (Geliebter and Aversa, 2003; Nolan et al., 2010).

### Internal Structure and Consistency (Sample 1, **Table 5**)

**Table 5:** Mean scores, reliability (internal consistency and test–retest stability) and Spearman’s correlation with BMI and DEBQ for each score of the EMAQ.

	Sample 1 (*n* = 604)			Sample 2 (*n* = 75)				
	Mean ±*SD* [range]	Cronbach’s Alpha	BMI	Mean ±*SD* [range]	ICC [95% CI]	DEBQ-EE	DEBQ-R	DEBQ-X
EMAQ-PE	5.15 ± 1.01 [1–9]	0.83	-0.162 (*p* < 0.000)	5.01 ± 0.63 [2.4–6.8]	0.482 [0.287–0.636]	-0.289 (*p* = 0.012)	-0.072 (ns)	0.147 (ns)
EMAQ-NE	4.69 ± 1.21 [1.2–8.9]	0.85	0.143 (*p* < 0.000)	5.13 ± 1.04 [2.4–7.8]	0.588 [0.418–0.719]	0.748 (*p* < 0.000)	0.081 (ns)	0.185 (ns)
EMAQ-PS	4.97 ± 1.20 [1–9]	0.35	-0.110 (*p* = 0.007)	4.90 ± 0.87 [3–8.5]	0.703 [0.567–0.802]	-0.191 (ns)	0.021 (ns)	-0.045 (ns)
EMAQ-NS	4.03 ± 1.36 [1–8.8]	0.70	0.193 (*p* < 0.000)	4.30 ± 1.18 [1.5–7.6]	0.758 [0.642–0.840]	0.547 (*p* < 0.000)	0.229 (*p* = 0.048)	0.032 (ns)
EMAQ-P	5.06 ± 0.95 [1–9]	0.75	-0.143 (*p* < 0.000)	4.95 ± 0.63 [2.9–7.2]	0.736 [0.612–0.825]	-0.317 (*p* = 0.006)	-0.034 (ns)	0.024 (ns)
EMAQ-N	4.36 ± 1.18 [1.1–8.6]	0.85	0.180 (*p* < 0.000)	4.72 ± 1.01 [2.3–7.4]	0.761 [0.646–0.842]	0.710 (*p* < 0.000)	0.121 (ns)	0.168 (ns)

*EMAQ, Emotional Appetite Questionnaire; PE, Positive Emotion; NE, Negative Emotion; PS, Positive Situation; NS, Negative Situation; P, Positive Total Score; N, Negative Total Score. BMI, Body Mass Index (kg/m^2^); ICC, Intra Class Correlation coefficient; DEBQ, Dutch Eating Behavior Questionnaire; EE, Emotional Eating; R: Restrained; X, External Eating.*

Pearson correlation between the EMAQ total positive score (EMAQ-P) and the EMAQ total negative score (EMAQ-N) was negative and significant (*r* = -0.130; *p* = 0.002). EMAQ Emotions and Situations subscale scores were positively significantly correlated (EMAQ-NE/EMAQ-NS: *r* = 0.685, *p* < 0.001; EMAQ-PE/EMAQ-PS: *r* = 0.482, *p* < 0.001).

Cronbach’s alpha coefficients for the EMAQ-P and EMAQ-N scores were 0.750 and 0.850 respectively.

### Test–Retest Reliability (Sample 2, **Table 5**)

The intra-class correlation coefficients for EMAQ-P and EMAQ-N were respectively 0.736 and 0.761.

### Convergent and Discriminant Validity (Sample 2, **Table 5**)

The EMAQ Negative scores and the DEBQ-EE were strongly positively correlated (EMAQ-N: ρ = 0.761, *p* < 0.001). Conversely, the EMAQ positive scores and the DEBQ-EE were moderately negatively correlated (EMAQ-P: ρ = -.317, *p* = 0.006).

### Associations with BMI (Sample 1, **Table 5**)

The EMAQ negative scores were modestly positively correlated with BMI (EMAQ-N: ρ = 0.180, *p* < 0.001), whereas the EMAQ positive scores were modestly negatively correlated with BMI (EMAQ-P: ρ = -0.143, *p* < 0.001).

### Between-Group Comparisons (Sample 1, **Table 6**)

**Table 6:** Emotional Appetite Questionnaire total and subscale scores according to gender and diagnosis, and *post hoc t-*test for EMAQ total scores (Sample 1, *n* = 604).

	Men (*n* = 242)	Women (*n* = 362)	*t*	*p*	*d*	Bulimia LT (*n* = 76)	No Bulimia LT (*n* = 528)	*t*	*p*	*d*
EMAQ-PE	5.38 ± 0.95	4.99 ± 1.01	–	–	–	4.87 ± 1.11	5.19 ± 0.99	–	–	–
EMAQ-NE	4.65 ± 1.00	4.71 ± 1.32	–	–	–	5.03 ± 1.55	4.63 ± 1.14	–	–	–
EMAQ-PS	5.13 ± 1.10	4.86 ± 1.26	–	–	–	4.53 ± 1.47	5.03 ± 1.15	–	–	–
EMAQ-NS	4.24 ± 1.16	3.89 ± 1.46	–	–	–	4.39 ± 1.71	3.97 ± 1.29	–	–	–
EMAQ-P	5.25 ± 0.85	4.93 ± 0.99	4.13	<0.000	0.35	4.70 ± 1.15	5.11 ± 0.91	3.51	<0.000	0.40
EMAQ-N	4.45 ± 0.98	4.30 ± 1.29	1.45	ns	0.13	4.71 ± 1.52	4.30 ± 1.11	-2.80	0.005	0.36

*EMAQ, Emotional Appetite Questionnaire; PE, Positive Emotion; NE, Negative Emotion; PS, Positive Situation; NS, Negative Situation; P, Positive Total Score; N, Negative Total Score; Bulimia LT, Bulimia Lifetime risk (CIDI screening).*

Seventy-six participants (54 women) presented a risk for BN. BMI differed significantly between men and women and between students at risk versus not at risk for BN (respectively Mean = 22.8; *SD* = 4.6 and Mean = 21.6; *SD* = 2.9; *p* = 0.003).

The analyses (BMI-adjusted) indicated a main effect of Gender [*F*(1,583) = 13.89; *p* < 0.001, $\eta_{p}^{2}$ = 0.023], of [Positive versus Negative] Valence [*F*(1,583) = 52.14; *p* < 0.000, $\eta_{p}^{2}$ = 0.082] and a significant Gender × Valence interaction [*F*(1,583) = 3.71; *p* = 0.050, $\eta_{p}^{2}$ = 0.006]. *Post hoc t*-test showed higher EMAQ-P scores among men, but no differences for the EMAQ-N.

Regarding the risk for BN, analyses (BMI-adjusted) indicated no main effect of Diagnosis [*F*(1,583) = 0.006; *p* = 0.972], a main effect of Valence [*F*(1,583) = 33.91; *p* < 0.001, $\eta_{p}^{2}$ = 0.055] and a significant Diagnosis × Valence interaction [*F*(1,583) = 12.81; *p* < 0.001, $\eta_{p}^{2}$ = 0.022]. *Post hoc t*-test revealed that students with a risk for BN had lower EMAQ-P but higher EMAQ-N scores.

## Discussion

The present study describes the French adaptation of the Emotional Appetite Questionnaire (F-EMAQ) and its validation among university students.

Exploratory factor analysis and CFA analyses revealed a two-factor structure reflecting two major dimensions in the F-EMAQ: the Positive versus Negative valence of the items, rather than the theorized Emotions versus Situations by valence (i.e., four-factor) structure. Our study is the first to have tested the factorial structure of the EMAQ. The original scoring scheme cannot be challenged based on the results of a single study and additional research is required, including in a clinical population, to determine the appropriateness and added value of using all the EMAQ subscale scores.

With regard to its psychometric properties, the F-EMAQ presents good reliability. The internal consistency indices were satisfactory and the Cronbach’s Alpha coefficients for the EMAQ-Positive and EMAQ-Negative total scores were above 0.70. Results also demonstrate adequate test–retest reliability for the Positive and Negative total scores.

In line with the findings by Nolan et al. (2010), the F-EMAQ showed satisfactory convergent and discriminant validity with the DEBQ-EE. Convergent validity was demonstrated by the positive association between the EMAQ negative scores and the DEBQ-EE scores, which reflect the same construct. In contrast, in favor of the discriminant validity of the scale, we observed negative associations between the EMAQ positive scores and the DEBQ-EE scores. Future studies, not only in larger and more diverse populations, but also with the EES-II, which includes positive emotions, should help to provide further arguments for the convergent and discriminant validity of the EMAQ.

In the present study, the positive association between EMAQ negative scores and BMI suggest that people who reported being prone to eating more in response to negative emotions and situations (emotional eaters) had higher BMI than non-emotional eaters. This is in line with the two previous validation studies (Geliebter and Aversa, 2003; Nolan et al., 2010). It also adds to the literature incriminating EE in overeating and the obesity epidemic (Gibson, 2012). In our study, despite the small number of the students at risk for a lifetime diagnosis of BN, we found they had higher BMIs than the others and also appeared more prone to EE in response to negative emotions and situations. These results are coherent with the affect regulation model of BN suggesting that patients binge-eat in order to reduce negative affect (Polivy and Herman, 1993). They are also consistent with those of Ricca et al. (2012) who showed that BN patients reported eating more in response to negative emotional states (evaluated with the EES) than healthy controls. Nonetheless, in their study no significant differences were found between patients with anorexia nervosa (either restrictive or binge-purging type) and BN, but all the patients reported higher EES scores than the healthy control group. Yet, using the DEBQ-EE subscale, Vervaet et al. (2004) highlighted a continuum of EE scores along the BMI spectrum, whereby patients with anorexia nervosa restrictive-type had the lowest scores, patients with anorexia nervosa binge-purging type had intermediate scores, and those with BN had the highest scores.

While one would expect that increasing food intake, no matter the reason, leads to weight gain, here we observed negative associations between BMI and EMAQ positive scores: students who reported eating more in response to positive emotions and situations had the lowest BMI. This replicates the findings of the two previous validation studies (Geliebter and Aversa, 2003; Nolan et al., 2010). Hence, eating in response to positive emotions or situations seems to be not associated with weight gain. Of interest too is the observation that the students at risk for a lifetime diagnosis of BN (who had high BMIs and EMAQ negative scores) also appeared to be less prone to eat in response to positive emotions and situations than the others. Our results are consistent with the raising idea that people who eat in response to negative emotions differ from those who eat in response to positive emotions (Bongers et al., 2013a,b) and add to the recent discussion that they may represent two different constructs (van Strien et al., 2013, 2016). Similar studies, but with the EMAQ, should help to better understand the relationships between different eating disorders, concerns about weight and eating behaviors and modes of modulating food intake in response to unpleasant as well as pleasant experiences.

Besides exercising, which was not measured here, another factor that could influence the links between eating and body weight concerns the type of food consumed in relation to the valence of emotions (pleasure or displeasure) as previous studies demonstrated that negative emotions increase the consumption of junk food and positive emotions increase the consumption of healthy foods (Lyman, 1982; Macht, 1999; Macht et al., 2002). Additional comparative studies of underweight, normal and overweight people, coupling the evaluation of both positive and negative EE with the EMAQ and the type of food consumed [using daily food diaries or ecological sampling assessments (Adriaanse et al., 2011; Haedt-Matt and Keel, 2011)] should help to increase our knowledge on this issue.

As in the two previous studies on the EMAQ (Geliebter and Aversa, 2003; Nolan et al., 2010), men had higher EMAQ positive scores than women. These results support the hypothesis that men may be more likely to eat comfort foods to maintain or enhance positive emotions than women (Dubé et al., 2005). However, in our study, men and women did not differ as regards eating more in response to negative situations. This result contrasts with what is traditionally reported in the literature about EE, which is that EE in response to negative states or situations is a more frequent characteristic of women’s eating behaviors (Gibson, 2012; Camilleri et al., 2014). To correctly interpret the effects of gender, we need first to determine whether the underlying psychometric properties of the EMAQ are invariant (i.e., equivalent) across gender (Gregorich, 2006). The same holds true for age. Thus if measurement invariance across gender/age does not hold, total score differences across gender/age groups are difficult to interpret, as they could result either from measurement differences or from genuine differences in emotionally driven eating behaviors. Accordingly, additional data are being collected to investigate this issue in a larger and more diverse sample.

The current study presents some limitations. First, it was conducted among students, so there are limits to the generalizability of the findings. Moreover, the range of academic disciplines represented was relatively restricted (and included many psychology students) and some factors that could influence EE, such as ethno-culture, religion, socio-economic background, incomes or the level of physical activity were not taken into account (Dubé et al., 2005; Diggins et al., 2015). As regards the biases inherent to self-report questionnaires, further attention should be devoted to how factors that are particularly critical in the area of weight control and eating behaviors, such as the ability for introspection or the social desirability bias, impact responding to the EMAQ.

## Conclusion

Today, EE is considered as a real risk factor for eating disorders and obesity. The literature and our findings highlight that, beyond their intensity, the valence of emotions has an influence on eating and potentially on weight. To few instruments assessing emotionally driven eating habits in French are available and validated. The EMAQ appears to be a promising instrument evaluating subjective variations in food intake (eating less, equally, or more) in response to positive and negative emotional states and situations. It could help clinicians to better understand the individual differences that could impact food intake and to tailor specific therapeutic interventions.

## Author Contributions

LK, LR, YM, and SB designed and performed the study. LB and SB conducted the literature searches. LB, CL, and SB undertook the statistical analyses. LB, CL, and SB wrote the first draft of the manuscript. All authors significantly participated in interpreting the results and revising the manuscript.

## Conflict of Interest Statement

The authors declare that the research was conducted in the absence of any commercial or financial relationships that could be construed as a potential conflict of interest.

## Footnotes

1 See also Bongers and Jansen (2016) for a discussion on the validity of self-reported emotional eating.

2 See Gibson (2012) and Bongers and Jansen (2016) for a full overview of EE questionnaires.
